# Polymorphisms in tumour necrosis factor (TNF) are associated with risk of bladder cancer and grade of tumour at presentation

**DOI:** 10.1038/sj.bjc.6601165

**Published:** 2003-09-09

**Authors:** H P Marsh, N A Haldar, M Bunce, S E Marshall, K le Monier, S L Winsey, K Christodoulos, D Cranston, K I Welsh, A L Harris

**Affiliations:** 1 Transplant Immunology Laboratory, Oxford Radcliffe Hospitals, Oxford OX3 7LJ, UK; 2 Department of Urology, Oxford Radcliffe Hospitals, Oxford OX3 7LJ, UK; 3 Imperial Cancer Research Fund Medical Oncology Unit, Oxford Radcliffe Hospitals, Oxford OX3 7LJ, UK; 4 Emanuel Kaye Building, National Heart and Lung Institute, Manresa Road, London SW3 6LR, UK

**Keywords:** TNF; polymorphisms; bladder cancer; grade

## Abstract

The purpose of this study is to assess the role of tumour necrosis factor (TNF) polymorphisms in the risk of developing bladder cancer and effect on tumour stage, grade and progression. In all, seven single-nucleotide polymorphisms in TNF were studied in 196 bladder cancer patients and 208 controls using a PCR-SSP genotyping technique. It was seen that there was a significant association of two polymorphisms in TNF with bladder cancer: the TNF+488A allele was found in 28.1% of patients compared with 14.9% of controls (*P*=0.0012). In addition, TNF−859T was found in 26.0% of patients compared with 14.4% of the controls (*P*=0.0036). The two loci were in tight linkage disequilibrium, that is, almost all the individuals having TNF+488A also had TNF−859T. Patients with the TNF+488A or TNF−859T were more likely to present with a moderately differentiated tumour than those patients without the uncommon allele. In all, 16.7% of patients with TNF+488A and 29.9% of patients without TNF+488A presented with a G1 tumour (*P*=0.015). A total of 14% of patients with TNF−859T and 30.5% of patients without TNF−859T presented with a G1 tumour (*P*=0.0043). There was no significant effect on time to first recurrence, stage progression or grade progression. In conclusion, a significant association between TNF polymorphisms TNF+488A and TNF−859T and risk of bladder cancer was detected in this study. Both these polymorphisms were associated with grade of tumour at presentation although there was no significant effect on subsequent tumour behaviour.

Transitional cell carcinoma (TCC) comprises 90% of all cases of bladder cancer. An estimated 57 400 new cases (42 000 men and 15 200 women) will be diagnosed with TCC in the USA in 2003. A total of 12 500 people (8600 men and 3900 women) will die of the disease (Jemal *et al*, 2003). In all, 70% present with superficial cancer, but of these 70% recur after treatment and 20–30% progress to invasive disease (Hassen and Droller, 2000).

Tumour necrosis factor alpha (TNF*α*) has been implicated in the development and progression of tumours (Naylor *et al*, 1992) and is known to be important in angiogenesis and is a key proinflammatory cytokine. In high doses, it acts to destroy tumour vasculature but in low doses its role is analogous to that in inflammation, acting as a vasodilator and chemoattractant for cells responsible for the inflammatory response and thus promoting the development of tumour stroma.

There is considerable direct and indirect evidence to implicate TNF*α* in the development of bladder cancer. TNF*α*-deficient mice have a greatly reduced skin tumour growth and tumour induction in response to chemical carcinogens (Moore *et al*, 1999). In angiogenesis, TNF*α* is known to play a key role in the regulation of the enzyme thymidine phosphorylase (Leek *et al*, 1998), which in turn has been demonstrated to be a component in bladder cancer progression (O'Brien *et al*, 1996). TNF*α* has also been demonstrated in bladder tumour biopsies by immunohistochemistry (Sander *et al*, 1996) and has been shown to be involved in both the local and systemic immune response to the intravesical instillation of bacille Calmette-Guerin (BCG) (Taniguchi *et al*, 1999). Following stimulation with BCG, increased levels of TNF*α* protein have been demonstrated in the urine of human patients (Taniguchi *et al*, 1999) and increased TNF*α* mRNA has been demonstrated in the bladders and spleens of mice (Shin *et al*, 1995). The importance of TNF*α* in bladder cancer pathogenesis raises the possibility that variation in the gene for TNF*α* (TNF) may predispose to risk of bladder cancer or alter subsequent tumour behaviour.

Tumour necrosis factor has been extensively investigated for genetic variation. The gene for TNF*α* is found on chromosome 6 in the class III region of the major histocompatibility complex (MHC) and is flanked by the lymphotoxin *α* and *β* genes. There are 16 known polymorphisms in and around TNF, including five microsatellites. The two most studied single-nucleotide polymorphisms (SNPs) are the promoter polymorphisms −308G/A and −238G/A. Other polymorphisms that may be of significance are +488G/A in the first intron, and the promoter variants, −1032T/C, −865C/A, −859C/T and −380G/A. There have been many efforts to determine the significance of these polymorphisms with extensive disease association and functional studies, the results of which have been conflicting (Allen, 1999; Bidwell *et al*, 1999).

One of the difficulties with finding an association with any SNP in the MHC region is the strong, but variable, linkage disequilibrium. At meiosis, a set of alleles on the same small chromosomal segment tends to be transmitted as one block, that is, a haplotype. As a result, any given allele may simply be a marker for another allele. In addition, an SNP at a given locus in one individual may be part of a haplotype that has an effect on protein expression or function, whereas exactly the same SNP in another individual may not form part of the functional haplotype and no functional effect is seen.

However, there is clear evidence for individual variation in TNF*α* production and considerable evidence supports the association of the −308 haplotype with high TNF*α* production (Wilson *et al*, 1993, 1997).

An association between malignancy and TNF polymorphisms includes non-Hodgkin's lymphoma (Warzocha *et al*, 1998) and myeloma (Davies *et al*, 2000). In addition, an association between two different polymorphisms, TNF+488, TNF−308, and prostate carcinoma has been demonstrated (Oh *et al*, 2000). Studies demonstrating an association with malignancy and TNF microsatellites include colorectal carcinoma (Gallagher *et al*, 1997) and laryngeal carcinoma (Matthias *et al*, 1998).

Given the key role of TNF*α* in angiogenesis and tumour development, the aim of this study was to investigate whether polymorphisms in TNF alter the risk of developing bladder cancer and subsequent tumour behaviour. We determined the frequency of these polymorphisms in bladder cancer patients and a control population, and correlated these results with the stage and grade of tumour and number of lesions at presentation and subsequent tumour recurrence and progression.

## METHODS

This prospective study was approved by the Central Oxford Research Ethics Committee. Consecutive patients with histologically proven bladder cancer attending the Urology Department, Churchill Hospital, Oxford between October 1999 and October 2000 were studied. At the time of recruitment, 5 ml of peripheral blood was taken in an EDTA tube for DNA. A clinical database was developed, including patients' demographic details, previous medical history and family history of cancer. Findings and treatment at initial presentation and subsequently throughout cystoscopic surveillance were recorded, with particular attention to number of lesions, stage and grade (see Table 1

**Table 1:** Clinical information for bladder cancer patients (*n*=196)

Age	
Median (range) (years)	68 (30–92)
Sex	
Male	151
Female	45
No. of lesions at initial presentation (*n*=186)	
1	137
2	26
3	12
>3	11
Stage at initial presentation (*n*=185)	
*cis*	5
a	54
1	89
2	33
3	4
Grade at initial presentation (*n*=191)	
G1	50
G2	92
G3	49

). All tumours were identified with cystoscopy. They then underwent transurethral resection and the specimens were examined by histopathologists. This allowed staging by the 1997 tumour node metastasis (TNM) classification. Complete clinical records were not available on 11 patients. In five patients, grade was not available, in 10 the number of lesions was not recorded and in 11 the stage was not available. All these patients were eligible for the analysis of polymorphism *vs* risk of cancer, but the cases that did not have full histological details were not included in the analysis for stage and grade and number of lesions.

**Figure 1:**
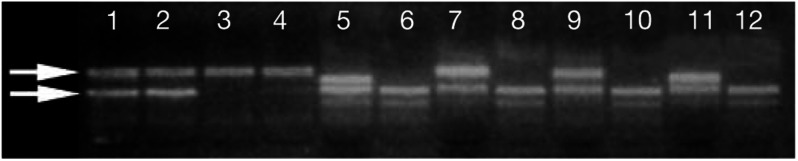
Polyacrylamide gel photograph showing TNF polymorphisms. Lanes 1–4 correspond to the four reactions used to identify the four previously documented haplotypes of the three biallelic polymorphisms, TNF+488, −238 and −308. The upper visible band is the control band. A positive reaction is seen in lanes 1 and 2, giving a result of TNF+488G, −238G, −308GA. Lanes 5–12 represent the remaining individual biallelic polymorphisms as follows: lanes 5 and 6: TNF−1032T,C; lanes 7 and 8: TNF−865C,A; lanes 9 and 10: TNF−859C,T; lanes 11 and 12: TNF−380G,A. In each case, the lower band is the control band. Positive reactions are seen in lanes 5,7,9 and 11 giving the result of TNF−1032T, −865C, −859C, and −380G.

DNA was extracted and PCR amplification using a unified polymerase chain reaction sequence-specific primers (PCR-SSR) genotyping technique was performed using protocols previously described (Bunce *et al*, 1995). Polymerase chain reaction products were then electrophoresed in 1% agarose gels and visualised with UV illumination, see Figure 1

. Seven SNPs in TNF: +488G/A, −238G/A, −308G/A, −1032T/C, −865C/A, −859C/T and −380G/A were analysed. To determine the haplotypes of the three most widely studied polymorphisms (TNF+488, −238, −308), we adapted a double amplification refractory mutation system (ARMS) method previously described (Fanning *et al*, 1997). This involves four primer mixes and from the pattern of bands in these reactions, the individual biallelic polymorphisms can be derived (Table 2

**Table 2:** PCR-SSP primer sequences, specificities and primer mix composition

	**Sense primer**	**Conc (mM)**	**Antisense primer**	**Conc (mM)**	**Amplicon size**
TNF+488G/−238G/−308G	5′-g C A T C C C C g T C T T T C T C C A C	0.51	5′ A T A g g T T T T g A g g g g C A T g g	0.51	835
TNF+488G/−238G/−308A	5′-g C A T C C C C g T C T T T C T C C A C	1.7	5′ A T A g g T T T T g A g g g g C A T g A	1.7	835
TNF+488G/−238A/−308G	5′-g C A T C C C C g T C T T T C T C C A C	0.85	5′ g A A g A C C C C C C T C g g A A T C A	0.85	763
TNF+488A/−238G/−308G	5′-g C A T C C C C g T C T T T C T C C A T	0.51	5′ g A A g A C C C C C C T C g g A A T C g	0.51	763
TNF−1032T	5′-C C g g g A A T T C A C A g A C C C C	3.4	5′-C A A A g g A g A A g C T g A g A A g A T	3.4	433
TNF−1032C	5′-C C g g g A A T T C A C A g A C C C C	3.4	5′-C A A A g g A g A A g C T g A g A A g A C	3.4	433
TNF−865C	5′-C C g g g A A T T C A C A g A C C C C	3.4	5′-C g A g T A T g g g g A C C C C C C	3.4	263
TNF−865A	5′-C C g g g A A T T C A C A g A C C C C	3.4	5′-g A g T A T g g g g A C C C C C A	3.4	262
TNF−859C	5′-C T A C A T g g C C C T g T C T T C g	5.1	5′-A A g g A T A A g g g C T C A g A g A g	5.1	270
TNF−859T	5′-T C T A C A T g g C C C T g T C T T C A	5.1	5′-A A g g A T A A g g g C T C A g A g A g	5.1	270
TNF−380G	5′-g g C T g g g T g T g C C A A C A A C	3.4	5′-C C T g C A T C C T g T C T g g A A g	3.4	396
TNF−380A	5′-g g C T g g g T g T g C C A A C A A C	3.4	5′-T C C T g C A T C C T g T C T g g A A A	3.4	397

).

A major problem of genetic association studies is the inability to confirm results in additional cohorts (Editorial, 1999). To address this, we initially examined a first set of 99 consecutive bladder cancer patients and compared gene frequencies with a control population of 107 cadaveric renal transplant donors. Subsequently, a further 101 controls and 97 bladder cancer patients were examined. The patients were examined only for the polymorphisms that showed a positive association in the first set that is, +488 (together with −238 and −308) and −859. The representative nature of the control cohort of the UK population has been previously described in HLA typing reports (Bunce *et al*, 1996).

In addition, a set of 91 individuals with histologically confirmed malignant melanoma was investigated for the same TNF polymorphisms. This group served as an additional control population with malignancy unrelated to the urinary tract.

The results were analysed with the statistical package Knowledge Seeker™ (Angoss software, Toronto, Canada).

## RESULTS

### Frequency of polymorphisms in patients and controls

The presence of TNF+488A was significantly higher in the first set of bladder cancer patients compared with controls. It also approached significance in the second set, remaining significant with the two sets combined. In the first set, TNF+488A was found in 13.1% of controls compared with 28.3% of patients (*P*=0.0068) and in the second set, in 16.8% of controls compared with 27.8% of patients (*P*=0.063, see Table 3

**Table 3:** Phenotype frequencies for bladder cancer patients and controls; first and second sets

	**First set controls (*n*=107)**		**First set patients (*n*=99)**			**Second set controls (*n*=101)**		**Second set patients (*n*=97)**		
TNF+488G	107	100.0%	99	100.0%		100	99.0%	95	97.9%	
TNF+488A	14	13.1%	28	28.3%	*P*=0.0068	17	16.8%	27	27.8%	*P*=0.063

TNF−238G	106	99.1%	99	100.0%		100	99.0%	97	100.0%	
TNF−238A	17	15.9%	3	3.0%	*P*=0.0018	4	4.0%	18	18.6%	*P*=0.0011

TNF−308G	106	99.1%	98	99.0%		97	96.0%	95	97.9%	
TNF−308A	33	30.8%	35	35.4%	*P*=0.49	36	35.6%	21	21.6%	*P*=0.0030

TNF−1032T	99	92.5%	97	98.0%		99	98.0%			
TNF−1032C	47	43.9%	36	36.4%	*P*=0.27	32	31.7%			

TNF−865C	105	98.1%	98	99.0%		100	99.0%			
TNF−865A	32	29.9%	26	26.3%	*P*=0.56	27	26.7%			

TNF−859C	107	100.0%	99	100.0%		99	98.0%	95	97.9%	
TNF−859T	14	13.1%	26	26.3%	*P*=0.017	16	15.8%	25	25.8%	*P*=0.085

TNF−380G	107	100.0%	99	100.0%		101	100.0%			
TNF−380A	4	3.7%	0	0.0%	*P*=0.05	1	1.0%			

The significant results TNF+488 and TNF−859 are highlighted with a grey background.

). With the two sets combined, TNF+488A was seen in 14.9% of controls and 28.1% of patients (*P*=0.0012, see Table 4

**Table 4:** Phenotype frequencies for combined controls, bladder cancer and melanoma patients

	**Controls *n*=208**			**Patients *n*=196/99**				**Melanoma *n*=91**			
TNF+488G	207	208	99.5%	194	196	99.0%		91	91	100%	
TNF+488A	31	208	14.9%	55	196	28.1%	*P*=0.0012	13	91	14%	*P*=0.89

TNF−238G	206	208	99.0%	196	196	100.0%		91	91	100%	
TNF−238A	21	208	10.1%	21	196	10.7%	*P*=0.84	8	91	8.80%	*P*=0.76

TNF−308G	203	208	97.6%	193	196	98.5%		90	91	99%	
TNF−308A	69	208	33.2%	56	196	28.6%	*P*=0.32	34	91	37%	*P*=0.48

TNF−1032T	198	208	95.2%	97	99	98.0%		89	91	98%	
TNF−1032C	79	208	38.0%	36	99	36.4%	*P*=0.78	35	91	38%	*P*=0.94

TNF−865C	205	208	98.6%	98	99	99.0%		89	91	98%	
TNF−865A	59	208	28.4%	26	99	26.3%	*P*=0.70	27	91	30%	*P*=0.82

TNF−859C	206	208	99.0%	194	196	99.0%		91	91	100%	
TNF−859T	30	208	14.4%	51	196	26.0%	*P*=0.0036	13	91	14%	*P*=0.98

TNF−380G	208	208	100.0%	99	99	100.0%		91	91	100%	
TNF−380A	5	208	2.4%	0	99	0.0%	*P*=0.12	5	91	5%	*P*=0.17

The significant results TNF+488 and TNF−859 are highlighted with a grey background.

).

The presence of TNF−859T was significantly higher in the first set of patients. In the second set, the presence was more frequent but did not reach significance. Nonetheless, significance was reached with the two sets combined. In the first set, TNF−859T was found in 13.1% of controls compared with 26.3% of patients (*P*=0.017) and in the second set, in 15.8% of controls compared with 25.8% of patients (*P*=0.085, see Table 3). With the two sets combined, TNF−859T was seen in 14.4% of controls and 26.0% of patients (*P*=0.0036, see Table 4). These results were not independent of each other as the TNF+488 and TNF−859 loci were in strong linkage disequilibrium, that is, almost all the individuals with TNF+488A also had TNF−859T.

No significant associations between the TNF polymorphisms and melanoma were detected (Table 4).

### Association with stage and grade at presentation

Information on grade of tumour at presentation was available on 191 patients. There was a significant association between the grade of tumour at initial presentation and the polymorphisms TNF−859T (*P*=0.0043) and TNF+488A (*P*=0.015, see Table 5

**Table 5:** Relationship between grade of tumour at presentation and TNF−859, TNF+488 polymorphism (information available on 191 of 196 patients)

	**−859T: yes**		**No**		**Population of 191 patients:**	
G1	(7)	14.0%	(43)	30.5%	(50)	26.2%
G2	(34)	68.0%	(58)	41.1%	(92)	48.2%
G3	(9)	18.0%	(40)	28.4%	(49)	25.6%
	50	26.2%	141	73.8%	191	
*χ*^2^ test −859T *vs* −859 not T, for G1, G2, G3 *P*=0.0043, for G1 *vs* (G2+G3) *P*=0.023						
	**+488A: yes**		**No**		**Population of 191 patients:**	
G1	(9)	16.7%	(41)	29.9%	(50)	26.2%
G2	(35)	64.8%	(57)	41.6%	(92)	48.2%
G3	(10)	18.5%	(39)	28.5%	(49)	25.6%
	54	28.3%	137	71.7%	191	
*χ*^2^ test +488A *vs* +488 not A, for G1, G2, G3 *P*=0.015, for G1 *vs* (G2+G3) *P*=0.06						

). Thus, those patients who had the uncommon allele at TNF+488 and TNF−859 were more likely to present with a moderately differentiated (Grade 2) tumour than those patients without the uncommon allele. Of 191 patients for whom clinical information was available, 50 (26.2%) presented with a G1 tumour, 92 (48.2%) with a G2 and 49 (25.6%) with a G3 (Table 5) tumour. Of the 50 patients with TNF−859T, seven (14%) presented with a G1 tumour. Of the 141 patients without TNF−859T, 43 (30.5%) presented with a G1 tumour. Although there were more G2 tumours in the TNF−859T group, there were fewer G3. *χ*^2^ tests were carried out for tumour distribution in three categories and also performed with G2 and G3 combined as a single group, G1 *vs* G2+G3. This showed that the distribution into the three categories separately was highly significant (*P*=0.0043) and there were also significantly more patients with G2+G3 *vs* G1 in the TNF−859T polymorphism group (*P*=0.023).

Of the 54 patients with the TNF+488A polymorphism, nine (16.7%) presented with a G1 tumour. Of the 137 patients who did not carry this variant, 41 (29.9%) presented with a G1 tumour. Again, *χ*^2^ tests were performed for tumour distribution in three categories and also performed with G2 and G3 combined as a single group. This showed that the distribution into the three categories separately was significant (*P*=0.015). When G2 and G3 were considered together, that is, G1 *vs* G2+G3, the association approached significance (*P*=0.06), see Table 5.

No association was found with polymorphisms and stage at presentation.

### Association with number of lesions at presentation

No association was found with polymorphisms and number of lesions at presentation.

### Association of TNF+488A and TNF−859T polymorphisms with prognosis

The log-rank test showed no association of TNF+488A or TNF−859T on time to first recurrence (TNF+488A, *P*=0.57, TNF−859T, *P*=0.41), time to first stage progression (TNF+488A, *P*=0.69, TNF−859T, *P*=0.79) or on time to first grade progression (TNF+488A, *P*=0.26, TNF−859T, *P*=0.12). The median time of follow-up from first diagnosis was 3.65 years. The range was 0.06–35.8 years.

## DISCUSSION

The role of TNF polymorphisms in the risk of bladder cancer, bladder cancer phenotype and subsequent tumour behaviour was assessed in this study. We have shown for the first time a significant association of the TNF polymorphisms TNF+488A and TNF−859T and risk of bladder cancer. The TNF+488A has been previously associated with rheumatoid arthritis (Kaijzel *et al*, 1998; Verweij, 1999) and common variable immunodeficiency (Mullighan *et al*, 1997) in addition to prostate cancer (Oh *et al*, 2000). At position TNF−859, no disease association studies have been reported and functional studies have been conflicting with Higuchi *et al* (1998) showing an effect on TNF transcription, while Uglialoro *et al* (1998) showed no effect.

In addition to the association of TNF+488A and TNF−859T with risk of bladder cancer, we have shown for the first time a very strong linkage disequilibrium between these two sites in bladder cancer patients, normal controls and melanoma patients. The linkage disequilibrium between TNF+488A and TNF−859T demonstrates the presence of a previously unreported haplotype; one that we hypothesise will have functional significance. Several TNF haplotypes have already been described involving TNF and the surrounding lymphotoxin gene (Fanning *et al*, 1997) and the surrounding HLA alleles (Wilson *et al*, 1993). The linkage between TNF+488A and TNF−859T that we have demonstrated may help to clarify further the role of polymorphisms in TNF and may explain the previously reported disease associations with the TNF+488 polymorphism, as TNF+488 is in the first intron of the gene and itself noncoding. Interestingly, Verweij (1999) demonstrated no effect of the TNF+488 polymorphism on TNF*α* production despite demonstrating a clear association with rheumatoid arthritis, suggesting that the disease associations are the result of linkage disequilibrium with alleles within or near the TNF/Lymphotoxin locus.

The mechanisms that could relate polymorphisms that upregulate inflammatory pathways to cancer are well recognised (Balkwill and Mantovani, 2001), including generation of free radicals (Marshall *et al*, 2000) and DNA damage (Winsey *et al*, 2000), enhanced angiogenesis and also immunosuppressive effects at higher concentrations. Chronic inflammation of the bladder as seen in schistosomiasis and long-term catheter use are well-recognised risk factors for bladder cancer (Locke, 1985; Stonehill, 1996) and inflammation associated with superficial bladder cancer is a common observation at operation.

In addition to their role in the risk of bladder cancer, we investigated the role of TNF polymorphisms in tumour behaviour. We have shown a significant association of both TNF+488A and TNF−859T with grade of tumour at presentation. If the presence of TNF+488A or TNF−859T affects the development of bladder tumours to make them generally less well differentiated, then we would expect to see more G3 as well as G2 tumours. It is possible that upregulation of TNF*α* or TNF*α*-dependent pathways direct the progression of bladder cancer to grade 2, but other additional factors are involved in progression from grade 2 to grade 3, in keeping with the evidence of stepwise progression of bladder cancer. For example, in other types of cancer, clear upregulation of oncogenes to cause a specific subtype of disease are recognised egg AML and ALL.

Finally, there was no association of the TNF+488A or TNF−859T polymorphisms with recurrence or progression. This type of result has been found in other cancer studies of TNF polymorphisms. For example, in non-Hodgkin's lymphoma, although TNF polymorphisms have been shown to influence treatment outcome, progression-free survival and overall survival of patients (Warzocha *et al*, 1998), in myeloma TNF and lymphotoxin *α* polymorphisms are associated with increased risk of the disease, but no overall increase in survival (Davies *et al*, 2000).

The natural history of G2 tumours is very heterogeneous and it is this group in particular that needs better prognostic information. A larger study would be needed to demonstrate whether TNF genetic variants influence prognosis within the G2 subgroup.

In conclusion, we have demonstrated for the first time a TNF polymorphism association with risk of bladder cancer and grade of tumour at presentation. This is a further urological tumour association involving the polymorphism at position TNF+488, which has already been shown to be associated with prostate cancer. We have also demonstrated an additional TNF haplotype, which we hypothesise is of functional significance and which may help clarify the role of SNPs in influencing TNF*α* protein expression. If it affects the responsiveness of TNF*α* production to toxic stress, then this may explain the relation to risk of bladder cancer and higher grade and determine the effectiveness of intravesical BCG. Furthermore, there are now specific inhibitors of TNF*α* such as etanercept (Spencer-Green, 2000) and infliximab (Sands, 1999) which could be investigated.

Further investigation will require a molecular approach analysing the gene promoter in bladder epithelium.
